# Dictating translations with automatic speech recognition: Effects on translators’ performance

**DOI:** 10.3389/fpsyg.2023.1108898

**Published:** 2023-01-19

**Authors:** Lulu Wang, Sanjun Sun

**Affiliations:** School of English and International Studies, Beijing Foreign Studies University, Beijing, China

**Keywords:** automatic speech recognition, input modality, keylogging, translation process, speech-to-text conversion, sight translation, cognitive effort

## Abstract

Technologies can greatly improve translators’ productivity and reduce their workload. Previous research has found that the use of automatic speech recognition (ASR) tools for dictating translations can increase productivity. However, these studies often had small sample sizes and did not consider other important aspects of translators’ performance, such as translation quality and cognitive effort. This study aims to investigate the impact of text input method on translators’ performance in terms of task duration, time allocation, editing operations, cognitive effort, and translation quality, as well as whether text difficulty affects these factors. To do this, 60 Chinese translation trainees were randomly assigned to either a dictation group or a typing group, and completed two English-Chinese translations of varying levels of source-text difficulty. Data were collected using keylogging, subjective ratings, screen recording, and a questionnaire. The results showed that using ASR reduced the typing effort of participants without negatively affecting translation quality, but did not save time or reduce cognitive effort. No effect of text difficulty was observed. Analysis of the revisions made by the dictation group and the results of the post-test questionnaire provide insights into how ASR systems can be optimized for translation purposes.

## 1. Introduction

The translation industry is constantly seeking ways to improve speed and quality, leading to the incorporation of various technological advancements into translation workflows (Bowker, 2002; Mossop, 2006; O’Brien, 2012). With the rapid advancement of speech technologies, dictation using automatic speech recognition (ASR), also known as speech-to-text conversion, has the potential to be an alternative input method to traditional keyboard and mouse. Oral input has been shown to be faster than typing (Hauptmann and Rudnicky, 1990; Bowker, 2002), making ASR a promising tool for translation workflows. A large body of literature has explored the integration of ASR into translation processes and the ways in which speech technologies can be effectively utilized in translation (Dymetman et al., 1994; Brousseau et al., 1995; Reddy and Rose, 2010; Mesa-Lao, 2014; Zapata Rojas et al., 2017; Teixeira et al., 2019; Ciobanu and Secarǎ, 2020).

The benefits of ASR for ergonomics in translators’ work environments have been well-recognized (Ciobanu, 2014, 2016; Ehrensberger-Dow and Massey, 2014; Zapata Rojas, 2014, 2016; Ehrensberger-Dow and Hunziker Heeb, 2016). The traditional translation profession has often involved sitting at a desk for long periods of time, which can lead to health problems and physical discomforts such as repetitive strain injury, neck or back stiffness, and eyestrain or vision problems from staring at a computer screen. However, advances in speech technologies in recent decades have provided translators with an alternative that allows for greater comfort and mobility. ASR input allows translators to maintain more comfortable positions and move around freely, potentially leading to greater physical relaxation than using a keyboard (Ciobanu, 2016). Despite the recognized benefits of ASR for ergonomics, it is crucial to further investigate whether using ASR for translation dictation enhances translators’ performance. This is important for the translation industry and translator training programs, and can provide insights into the cognitive processes of translation.

## 2. Review of related literature

In the translation industry, it is often thought that the pursuit of higher productivity and translation quality is incompatible with reduced cognitive effort. In other words, when translators try to achieve higher translation quality in a shorter period of time, the effort they put into translation typically increases (Lacruz, 2017). Therefore, it is a question whether using ASR can optimize translators’ workflow by improving translation efficiency and quality without requiring additional effort. This section will review the relevant literature on these three aspects.

### 2.1. Productivity gains in ASR-assisted translation

ASR software can automatically transcribe spoken input into text (Ciobanu, 2014). With the help of ASR, translators can dictate their translations into a word processor, which is similar to sight translation in that both involve verbalized outputs (Mossop, 2006; Zapata Rojas, 2016). The main difference between dictated translation and sight translation is the additional review and editing phase in dictated translation to correct errors generated by the ASR tool and polish the dictated draft (Agrifoglio, 2004; Dragsted and Hansen, 2009). In addition, ASR-assisted translation allows for more flexibility in reading the source text, planning problem-solving strategies, and even rehearsing the translation, unlike in sight translation where these activities are more restricted (Shreve et al., 2011). Translators also do not need to focus on smooth delivery as much in ASR-assisted translation as they do in sight translation (Chen, 2015).

Sight translation can be four times as efficient as written translation (Dragsted and Hansen, 2009). This suggests that dictating a translation could be significantly faster than typing it if preparation time is included in task duration and revision time is excluded. However, when using ASR for text input, the time saved in initial composition would be used for later proofreading (Bowker, 2002; Vidal Ruiz et al., 2006; Ciobanu, 2016). Dragsted et al. (2011) took error correction time into account and found that dictation with ASR took more than twice as long as sight translation. This means that the revision stage took more than half of the total task duration when using ASR. Despite this, they found that dictating a translation using ASR was 24% faster than typing.

The productivity increase from using ASR has not only been observed in laboratory settings but has also been supported by translators’ experiences in real working conditions. Respondents to an online survey conducted by the University of Leeds’ Center for Translation Studies reported an average productivity increase of 110.56% when using this input mode compared to keyboard input (Ciobanu, 2014, 2016). However, there was a large individual variation in the data. While some respondents reported a five-fold increase in productivity, half of the respondents claimed a productivity increase of less than 35%. In general, most professional translators who had experience dictating their translations with ASR reported significantly higher productivity rates.

Previous research on ASR-assisted translation has primarily focused on Indo-European languages such as French, English, Spanish, and Danish (Dragsted and Hansen, 2009; Dragsted et al., 2011; Mees et al., 2013; García Martínez et al., 2014). However, Carl et al. (2016) found that dictation with ASR was also more efficient than typing for English-Japanese translation. They also noted significant differences in translation efficiency, pausing structure, translation units and segments, and time allocation in different stages of translation when translating from English into other European languages versus non-European languages like Hindi and Chinese. It is currently unclear if the productivity gains associated with ASR input can be observed in language pairs like English-Chinese.

Productivity gains from using ASR may vary depending on the recognition accuracy of the ASR system. ASR systems can have different levels of accuracy for different languages and may struggle with languages that have extensive use of elision or liaison (Bowker, 2002; O’Shaughnessy, 2008; Ciobanu, 2016). For example, respondents to the Leeds survey reported that dictating in French required more revision than in English, possibly due to a larger number of homophones and silent letters in French, which can lead to more spelling mistakes made by ASR systems (Ciobanu, 2016). Additionally, developing speech-to-text systems for Chinese, a tonal language with a high number of homophones, can be challenging (He, 2002; Wang et al., 2022). According to Désilets et al. (2008), when translators reach an average word error rate of 11.7% with ASR support, they may not experience significant productivity gains. However, when the word error rate in dictation is 4% or lower, users may achieve a productivity increase of around 35%.

### 2.2. Influences of ASR support on translation quality

Researchers have raised concerns about the potential impact of ASR on the style of translations produced orally (Mossop, 2006; Ciobanu, 2014). For example, Mossop (2006) argued that the use of ASR for dictation may result in more colloquial target texts, such as the use of more coordinate and fewer subordinate sentence structures. In line with this prediction, several Leeds respondents reported that their dictated target texts tended to be more informal and used simpler, more general language compared to their typed translations. This shift in language style may be due to a tendency to lower the register while dictating (Ciobanu, 2014).

Another potential issue with ASR input is that it may lead to lower translation quality due to misrecognition. Similar to typos that can occur when texts are produced using the keyboard, ASR software may produce errors known as “speakos” due to misrecognized homophones (Ciobanu, 2014, p. 535). Other types of errors that may occur with ASR input include filled pauses such as “um” and “uh” (Dragsted et al., 2011). Some of these errors may be difficult to detect, which can reduce the overall quality of the translation and the translator’s productivity as a thorough revision may be time-consuming.

However, the use of ASR in translation has also been suggested to improve translation quality in some cases. For example, in Désilets et al. (2008), participants reported that using ASR put them in a different mental state that resulted in better translations. Additionally, some respondents to the Leeds survey found it easier to identify unnatural expressions like translationese when using ASR (Ciobanu, 2016). Empirical data have also supported these claims. Dragsted and Hansen (2009) found no significant difference in the quality of professional translators’ sight and written translations. The results of Mees et al. (2013) showed even better quality of dictated translations with ASR than typed translations when error correction was in place. They suggested that speaking translations out loud could encourage translators to pay attention to larger units, and thus they would not be constrained to the word level.

### 2.3. Translators’ cognitive effort in the dictation mode

Few studies have specifically examined the cognitive effort required for ASR-assisted translation. Carl et al. (2016) found that ASR-assisted translation required slightly less effort than typing it based on the gazing and pause behavior of English-Japanese translators.

Several studies have compared the cognitive effort of written translation to that of sight translation (Jiménez Ivars, 2008; Dragsted and Hansen, 2009; Shreve et al., 2011). ASR-assisted translation involves a process similar to sight translation, so these findings may provide insights into the cognitive effort required for translation dictation. Jiménez Ivars (2008) experimentally studied English-to-Spanish sight translation and argued that it requires deeper cognitive processing than written translation due to the various psycho-physiological components it involves. Shreve et al. (2011) suggested that sight translation requires a high level of cognitive load due to the compressed time available for translators to understand, translate, and deliver the text. Agrifoglio (2004) even argued that the cognitive demands of sight translation are “by no means less” than those of simultaneous and consecutive translation (p. 43). However, Dragsted and Hansen (2009) found lower cognitive effort in English-to-Danish sight translation based on fixation counts, fixation duration, and pauses. Overall, research on the relative cognitive effort of sight translation compared to written translation is inconclusive, but most researchers agree that sight translation is more demanding in several ways.

Despite the similarities between sight translation and ASR-assisted translation, there are notable differences, such as the pressure to deliver fluently and the amount of planning time allowed. Therefore, previous research on sight translation cannot be seen as definitive evidence on the cognitive effort required for ASR-assisted translation. It is also worth noting that the cognitive effort required for sight translation can vary depending on factors such as the language pair being translated, the direction of translation, and the skills of the translator (Korpal, 2012; Su and Li, 2019; Su, 2020).

In summary, previous research on ASR-assisted translation has primarily focused on European language pairs and has largely examined the impact on productivity. However, these studies have not paid sufficient attention to other factors such as translation quality and cognitive effort, or the specific conditions that would make ASR a preferred choice over keyboard input. It is worth noting that the impact of source text features on translators’ performance with ASR systems has not been fully explored. Anecdotal evidence suggests that ASR-assisted translation may be more effective for free-flowing speech, but may not bring clear advantages in terms of saving effort for more complex texts (Ciobanu, 2016). Additionally, if translators fail to adequately organize their thoughts before dictating or hesitate during the process, the resulting draft may require a large number of edits, which could negatively impact productivity (Bowker, 2002). Further research is needed to determine the extent to which text difficulty, language pair, and other factors may influence the cognitive effort required for ASR-assisted translation.

The purpose of this study is to examine the effects of ASR assistance on English-Chinese translation performance in terms of task duration, time allocation, editing operations, cognitive effort, and quality. It also aims to explore translators’ perceptions of the integration of ASR into their workflow. To achieve these objectives, the following research questions were addressed: (1) How does ASR input influence translators’ time-on-task and their time allocation during the translation process? (2) Do translation quality, editing operations, and cognitive effort differ between typing and ASR input modes? (3) Does text difficulty modulate the effects of ASR input on translators’ performance, if any? (4) What are translators’ attitudes toward ASR-assisted translation and how can ASR tools be better tailored for translation purposes? This study focuses specifically on English-Chinese translation and considers the potential impact of source text difficulty on the use of ASR assistance.

## 3. Materials and methods

### 3.1. Participants

The study recruited 60 master’s students (51 female, 9 male) enrolled in the English-Chinese translation and interpreting program at Beijing Foreign Studies University (BFSU). Their ages ranged from 21 to 30 (*M* = 23.8, SD = 1.67). All participants had Chinese as their first language and English as their second language. They had received training in sight translation and were proficient touch typists. All procedures in the study were conducted in accordance with BFSU’s ethical standards for research involving human participants, and all participants provided informed consent.

### 3.2. Materials

The materials used in this study included two English source texts, a rating scale, and a post-translation questionnaire.

#### 3.2.1. Two source texts

For this study, two general English source texts were selected for translation. Text 1 was an excerpt from the children’s book *Unsolved! History’s Mysteries*, while Text 2 was taken from an article in *The Economist*. In order to evaluate translators’ performance under different input modes and levels of difficulty, both texts were matched in length but had distinct levels of difficulty. To measure the difficulty of the texts, the Flesch Reading Ease formula and the Flesch–Kincaid Grade Level test were used, as they have been previously found to be helpful in assessing translation difficulty (Jensen, 2009). However, it should be noted that readability scores do not provide conclusive evidence of translation difficulty (Sun and Shreve, 2014). Therefore, the difficulty levels of the source texts were also estimated by human evaluators, who were five students from the master’s programs in translation at BFSU.

Text 1 had a Flesch reading ease score of 75.1 and a Flesch–Kincaid grade level of 4.5, indicating that a fourth-grade US student would find it easy to understand. Text 2 had a Flesch reading ease score of 43.9 and a Flesch–Kincaid grade level of 10.3, meaning that for a native US English speaker, it would take 10 years of school education to fully understand it. Table 1 compares the features of the two texts.

**Table 1:** Readability statistics of the two source texts.

Source text	Count		Average length		Flesch reading ease	Flesch–Kincaid grade level
	Word	Sentence	Word	Sentence		
I	106	15	4.4	7	75.1	4.5
II	104	8	4.9	13	43.9	10.3

The five translation trainees were asked to rate their perceived difficulty of translating these two texts into Chinese on a 100-point scale, with 1 representing “extremely low translation difficulty level” and 100 standing for “extremely high translation difficulty level.” Their average ratings for Text 1 and Text 2 were significantly different, at 34 and 56, respectively (*p* < 0.05). Based on these results, we determined that Text 2 was more difficult to read than Text 1 and that translating these two texts would likely require different amounts of effort.

#### 3.2.2. Rating scale and questionnaire

In order to gain a more comprehensive understanding of cognitive effort during translation, a rating scale was used in addition to measures such as pauses. The NASA Task Load Index, adapted for translation difficulty by Sun and Shreve (2014), was utilized in this study, including four dimensions: mental demand, effort, frustration level, and performance. Participants rated these dimensions on a 10-point scale. In addition, participants in the dictation group were asked to complete a post-task questionnaire to provide insights into their experience with ASR systems and their opinions on integrating ASR into their translation workflow.

### 3.3. Experimental design and procedure

The study was conducted in May 2022, during which data on keylogging, screen recordings, ratings on text difficulty, responses to the post-task questionnaire, and translation outputs were collected. Two mainstream ASR systems in China were initially tested for accuracy and usability by a group of five students, who ultimately preferred the free, speaker-independent, cloud-based ASR system, Sogou Pinyin.^1^ This system was then used in the study.

The 60 participants were randomly assigned to either a dictation group or a typing group. All tasks were completed within the word processor of the keylogger Translog-II (Carl, 2012), which recorded participants’ keystroke activities, while the computer screen was recorded using the screen recorder EVCapture.^2^ The source text was displayed in the left window of the screen, and the target text was to be produced in the right window using either the ASR system or traditional keyboard input. The dictation group dictated a draft of their translation and made revisions using keyboard and mouse until they were satisfied with the final target text. They were given no time limit and were told that they could pause their dictation at any time.

In order to eliminate potential confounding variables, all participants were asked to translate both Text 1 and Text 2 into Chinese using the same computer, and the order of the texts was counterbalanced to ensure that any effects were not due to the order in which they were translated. To ensure the validity of the results, participants were not permitted to use online resources or consult dictionaries during the translation process (Carl et al., 2011). However, the meanings of any words that might cause difficulties for them were provided on paper.

At the beginning of each data collection session, we gathered some information about the participant, including their name, gender, age, and major. We then provided them with instructions for the task. To account for the potential influence of unfamiliarity with the computer’s keyboard on task time, we allowed participants to become accustomed to it by typing a short Chinese passage. Additionally, we provided extra time for the dictation group to familiarize themselves with the ASR software and practice using it with a short Chinese text.

Upon completing each of the two translations, participants were asked to provide ratings of their perceived difficulty of the text using the previously described rating scale. In addition, those in the dictation group were asked to complete a questionnaire upon finishing the two texts.

### 3.4. Data processing

To analyze the data, we used the management tool of CRITT Translation Process Research Database (TPR-DB)^3^ to process the log files and extract relevant features. The alignment of the source and target texts at sentence level was adjusted and verified using the Yet Another Word Alignment Tool (YAWAT) (Germann, 2008).

Human translation is generally described as involving three phases: an initial orientation or skimming phase, a drafting phase, and a revision phase, though the translator may not necessarily progress through the phases in a linear fashion (Jakobsen, 2003; Englund Dimitrova, 2005, 2010; Carl et al., 2011; Dam-Jensen and Heine, 2013; Dragsted and Carl, 2013). In order to compute the lengths of these three phases in each session, we identified key timestamps using the TPR-DB tables, screen recordings, and log files. To examine the revision of dictated drafts in the dictation condition, we compared the drafts and corresponding final translations of the dictation group using Microsoft Word.

The numbers of Chinese characters inserted and deleted using keyboard-and-mouse were compared between the dictation and typing groups to analyze the editing efforts of translators in different input modes. Characters entered through speech recognition were not included in the analysis, as the ASR tool went through several rounds of automatic error correction before completing the recognition of a meaning unit. However, Translog-II recorded these as insertions and deletions made by the participant, which resulted in some noisy data that were excluded from the analyses.

Pauses are often considered a reliable indicator of cognitive effort in language production and translation processes (Schilperoord, 1996; Lacruz and Shreve, 2014; Vieira, 2016; Lacruz, 2017). However, there is disagreement on the appropriate threshold for pauses to accurately reflect translation effort (Muñoz Martín and Cardona, 2019). Zhang (2020) highlighted the need to consider language pair and translation direction when determining pause thresholds. Based on Zhang’s (2020) extensive analysis of pauses in the English-Chinese translation process, we set the pause baseline at 1500 ms, meaning that we only considered intervals between two consecutive dictation or typing units that exceeded 1,500 ms. Pause lengths above this threshold were calculated in all translation sessions.

To evaluate the quality of the final translation outputs, three translation teachers were invited to conduct a blind holistic assessment. The evaluation was based on a 100-point scale and followed the criteria used for grading translation in Test for English Majors-Band 8 (TEM-8), a large-scale and high-stakes test administered by China’s Ministry of Education (see, e.g., Xiao, 2015). In order to ensure a high level of interrater reliability, five translation outputs of Text 1 and five of Text 2 were randomly selected for pre-grading. During this phase, the evaluators discussed and adjusted their grading. The interrater reliability was checked by calculating the intra-class correlation coefficient (ICC) values using R. The ICC estimate for the quality scores of Text 1 and Text 2 was 0.82 and 0.87, respectively, indicating a “good” level of reliability. The final quality score was the average of the scores given by the three evaluators.

Before conducting statistical analyzes in SPSS (Version 26), we checked the distribution of the data. With the exception of difficulty ratings and quality scores, all data did not follow a normal distribution. Therefore, we used the aligned rank transform, a robust non-parametric method, to calculate main and interaction effects (Wobbrock et al., 2011). The data were first aligned and ranked using ARTool^4^ and then analyzed using full factorial analysis of variance. For difficulty ratings and quality scores, we followed the same procedure without pre-processing the data.

## 4. Results

### 4.1. Task duration

We used an alpha level of 0.05 for all statistical tests. A mixed-model ANOVA on the aligned and ranked task duration showed no significant main effect of input mode, *F*(1, 58) = 2.61, *p* = 0.112, indicating no significant difference in task duration between the dictation group (Mdn = 770,477 ms) and the typing group (Mdn = 687,930 ms). We observed a significant main effect of text difficulty, *F*(1, 58) = 95.13, *p* < 0.001, indicating that the duration of translating Text 2 (with a higher difficulty level than Text 1; Mdn = 832,672 ms) was significantly longer than Text 1 (Mdn = 665,766 ms) in both input modes. The interaction effect was non-significant, *F*(1, 58) = 0.23, *p* = 0.635.

### 4.2. Time allocation

#### 4.2.1. Orientation phase

**Figure 1:**
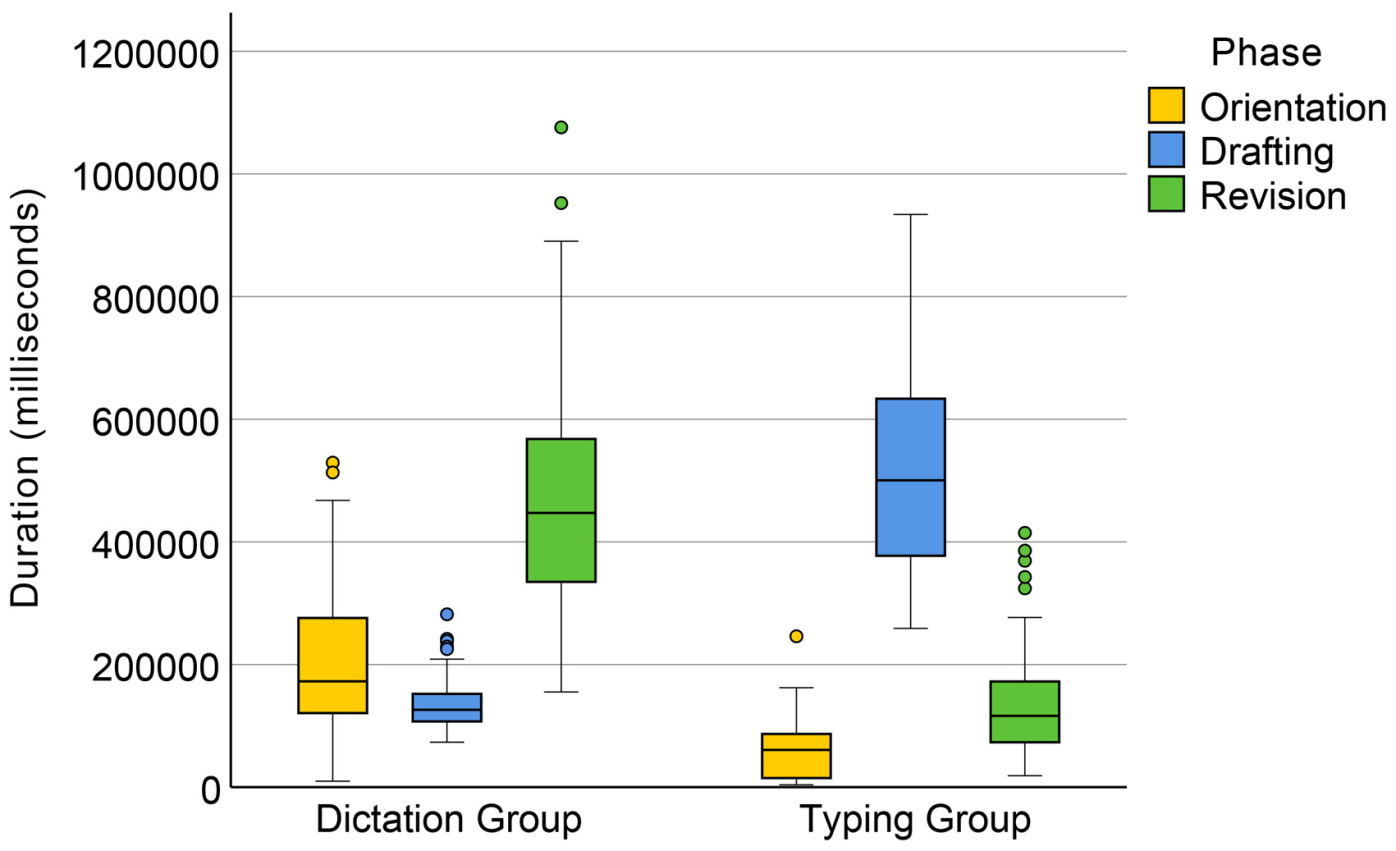
Time allocation in dictation and typing conditions.

**Figure 2:**
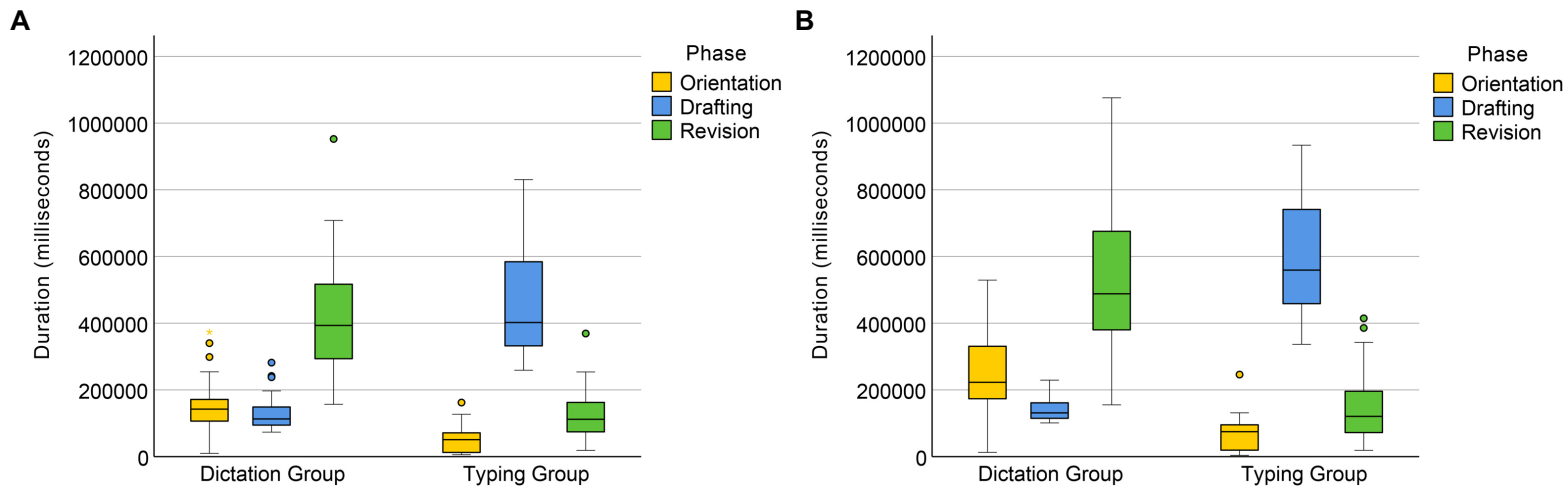
Time allocation in the translation of Texts 1 **(A)** and 2 **(B)**.

A Mann–Whitney U test on the original data of orientation duration revealed that regardless of text difficulty, the orientation time of the dictating group (Mdn = 172547.5 ms) was significantly longer than that of the typing group (Mdn = 60695.5 ms), *U* = 277, *p* < 0.001 (see Figure 1). A factorial ANOVA on the aligned and ranked data revealed a significant main effect of text difficulty, *F*(1, 58) = 56.12, *p* < 0.001. There was a significant interaction between input condition and text difficulty, *F*(1, 58) = 26.97, *p* < 0.001, indicating that the effect of text difficulty was greater in the dictating condition than in the typing condition (see Figure 2).

#### 4.2.2. Drafting phase

The drafting phase of the translation process was significantly shorter for the dictation group (Mdn = 126187.5 ms) compared to the typing group (Mdn = 500,375 ms), as indicated by a Mann–Whitney *U* test (*U* = 1, *p* < 0.001; see Figure 1). A factorial ANOVA on the aligned and ranked data for drafting time also revealed a significant main effect of text difficulty, *F*(1, 58) = 64.08, *p* < 0.001, and a significant interaction between input mode and text difficulty, *F*(1, 58) = 46.34, *p* < 0.001. The effect of text difficulty on drafting time was greater for the typing group than for the dictating group.

#### 4.2.3. Revision phase

Results from a Mann–Whitney U test showed that the dictation group (Mdn = 447,174 ms) had significantly longer revision times than the typing group (Mdn = 116250.5 ms), *U* = 144, *p* < 0.001 (see Figure 1). A factorial ANOVA on the aligned and ranked data revealed a significant main effect of text difficulty, *F*(1, 58) = 16.25, *p* < 0.001. There was also a significant interaction between input condition and text difficulty, *F*(1, 58) = 9.03, *p* = 0.004, indicating that text difficulty had a greater effect on revision times for the dictating group compared to the typing group (see Figure 2).

**Figure 3:**
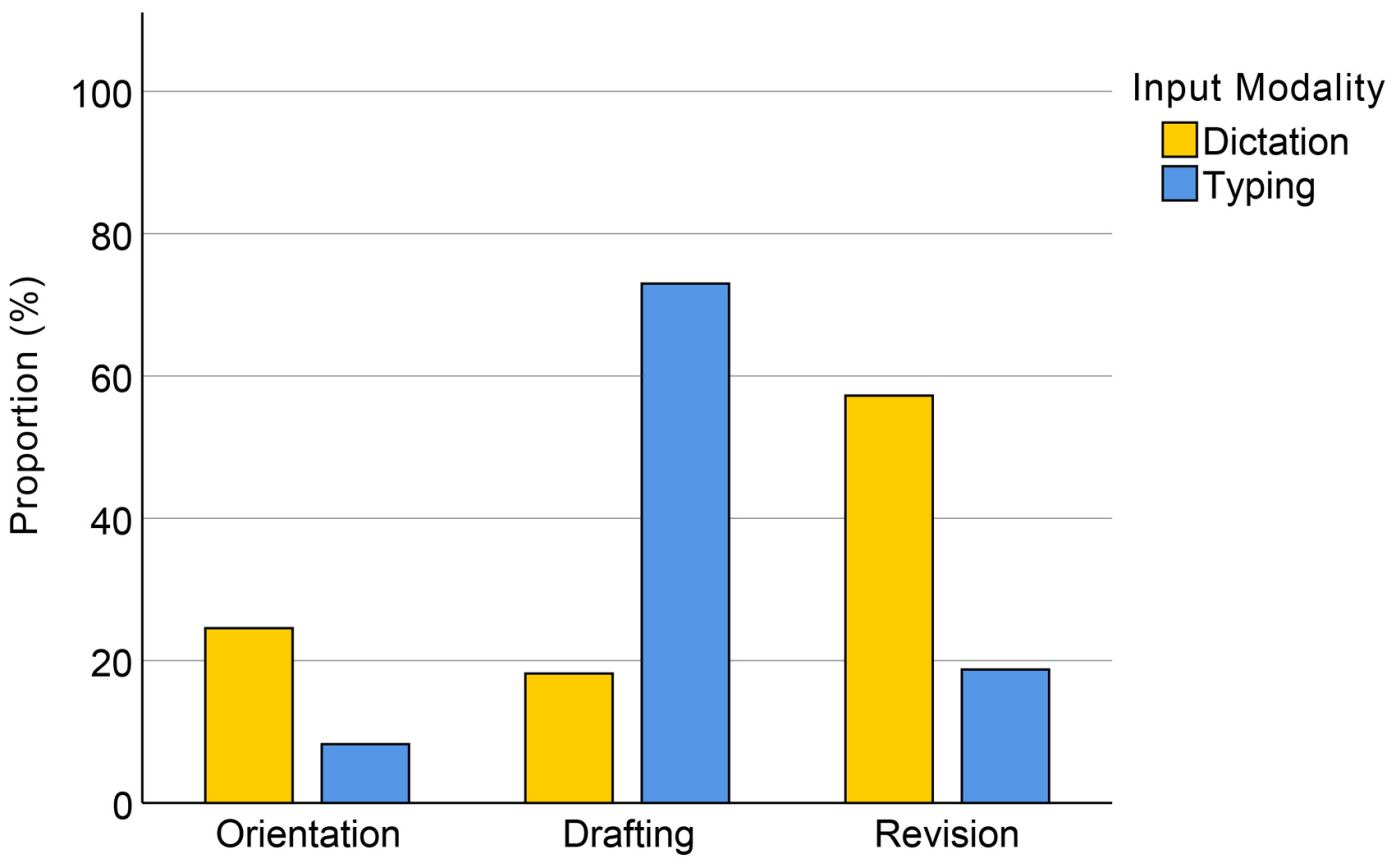
Proportions of the duration of each phase of the dictation group and the typing group.

The proportions of the three phases in total task time for the dictation and typing groups are presented in Figure 3. It can be seen that the dictation group spent approximately 60% of their total task time revising after completing their draft using ASR input, while the typing group devoted the majority (72.57%) of their time to the drafting phase.

In order to analyze the types of revisions made by the dictation group to their initial dictated drafts, we classified the revisions into four categories: wording, punctuation marks, ASR misrecognitions, and filled pauses (examples can be found in Table 2). The number of revisions in each category was calculated by first identifying the revisions made to each draft and then segmenting them at the phrase level. On average, over half of the editing operations made by the dictation group (54.21%) were rewordings of their dictated translations. The remaining revisions were devoted to correcting automatically generated punctuation marks (27.72%), correcting ASR misrecognitions (14.29%), and deleting filled pauses (3.79%).

**Table 2:** Examples of revisions made by the dictation group during the revision phase.

Type of revision	Example
Wording	飞机起飞后库珀先生。将一张纸条交给飞机乘务员
	这个。这种现象很十分奇怪
Punctuation	纸条上面写着。:
	经济发展最大的障碍经常是资源匮乏。，但并不总是这样。
Misrecognition	我现在要结机劫机了
	在哪加纳遇到了一个令人好奇的问题
Filled pause	他嗯递了一张纸条给成。记住，机组乘务员
	嗯，嗯。根据加纳政府与发电剬司签署的了一些非透明合同

**Figure 4:**
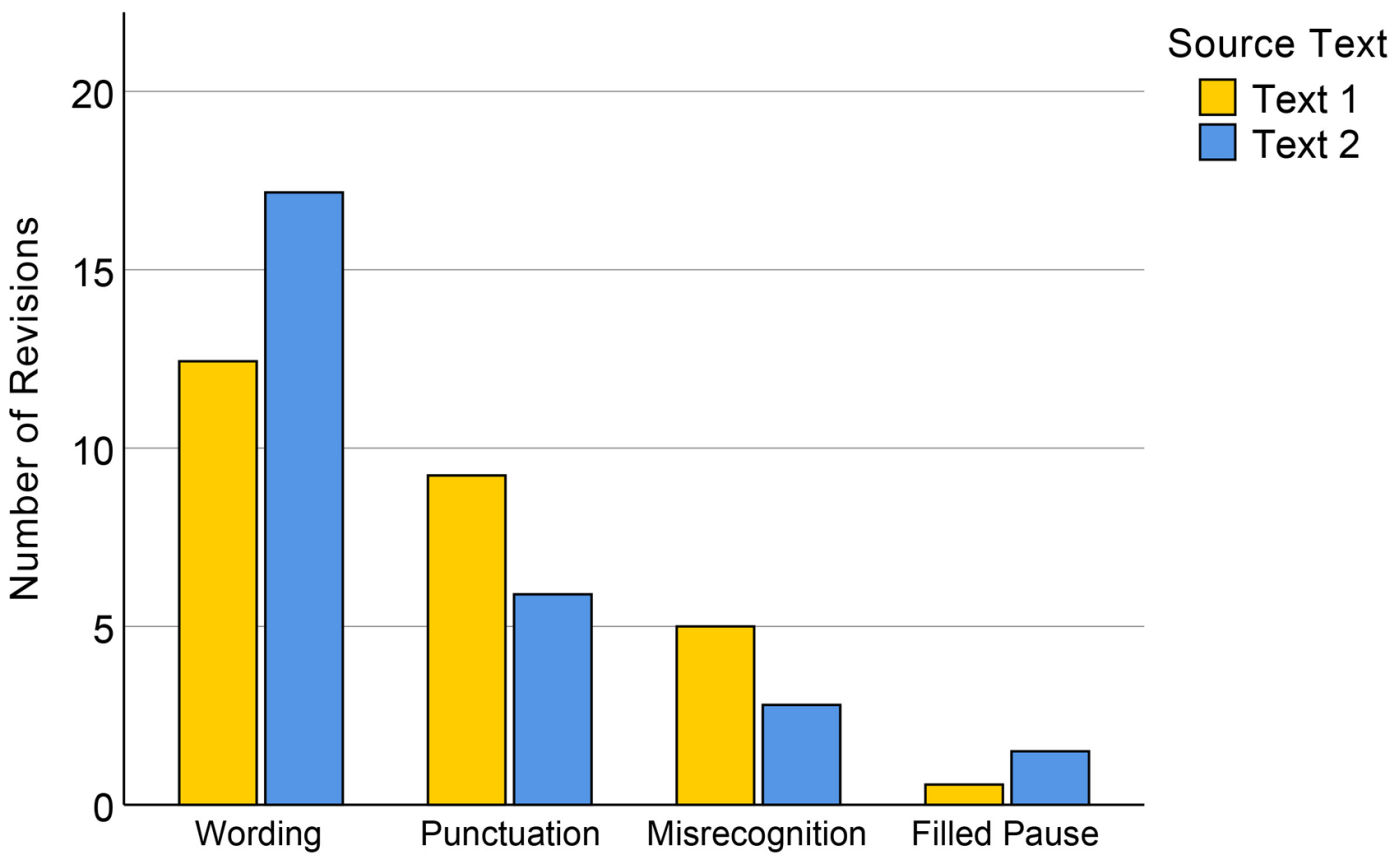
Comparison of revisions made by the dictation group in Texts 1 and 2.

The revision categories of the dictation group were compared across Text 1 and Text 2 (see Figure 4). A paired *t*-test on the numbers of revised wordings and punctuation marks showed significantly more wording changes in Text 2 (*M* = 17.17, SD = 5.24) than in the easier Text 1 (*M* = 12.43, SD = 5.14), *t*(29) = −5.07, *p* < 0.001. Conversely, the number of revised punctuations in Text 1 (*M* = 9.23, SD = 3.34) was significantly larger than in Text 2 (*M* = 5.90, SD = 2.56), *t*(29) = 5.04, *p* < 0.001. We used the Wilcoxon signed-rank test to compare participants’ revisions to misrecognitions between the two texts, as the data showed a non-normal distribution, with a significantly larger number for Text 1 (Mdn = 5) than Text 2 (Mdn = 3), *T* = 50, *Z* = −3.64, *p* < 0.001. The Wilcoxon test on the numbers of deleted filled pauses also showed a significant difference between the two texts, *T* = 143.5, *Z* = −2.62, *p* = 0.009.

### 4.3. Inserted and deleted characters

#### 4.3.1. Insertions

**Figure 5:**
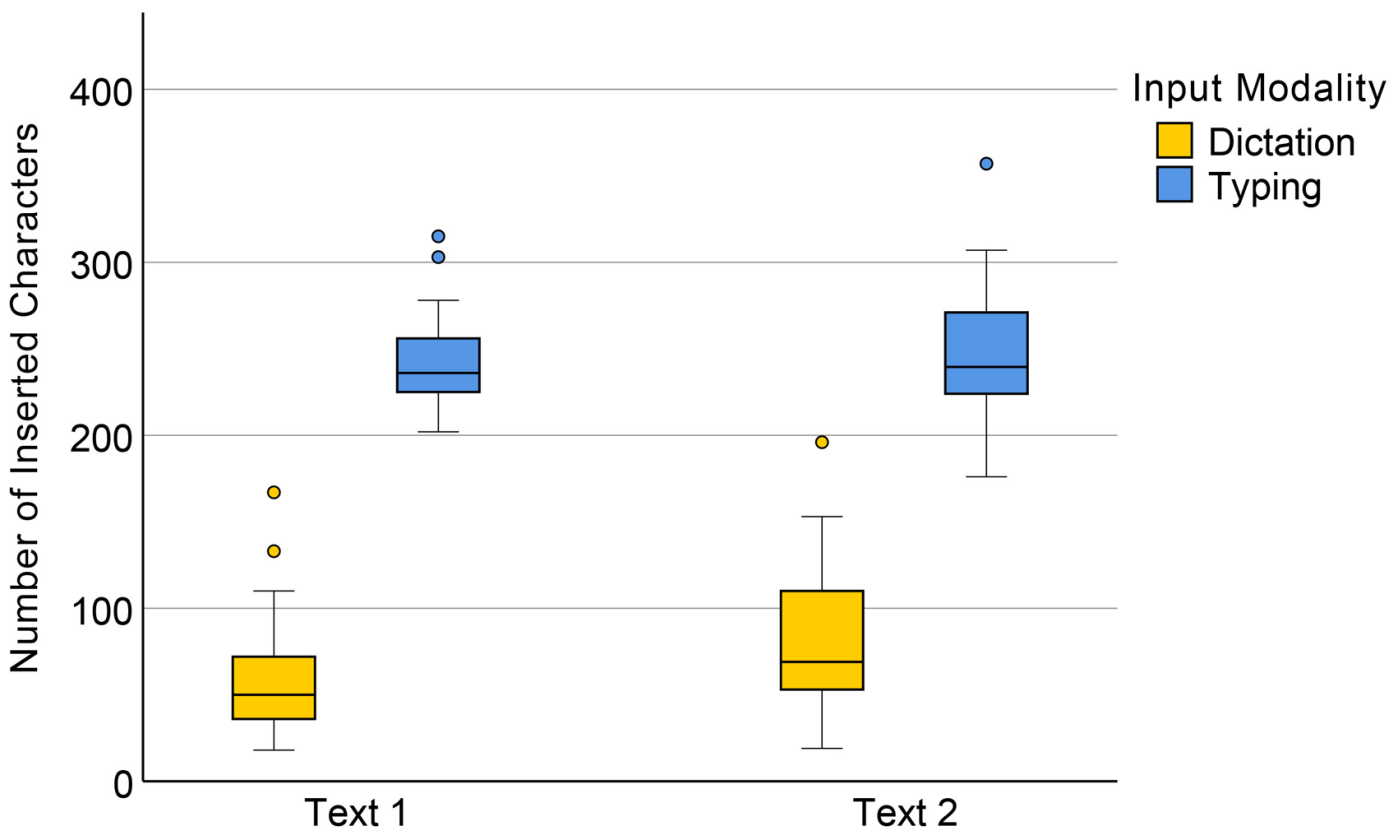
Numbers of inserted characters in dictation and typing conditions.

For the number of inserted characters, a significant main effect of input mode was observed, *F*(1, 58) = 142.02, *p* < 0.001, suggesting that the typing group made significantly more insertions than the dictation group, as illustrated in Figure 5. There was also a significant main effect of text difficulty, *F*(1, 58) = 7.99, *p* = 0.006. The interaction effect between input mode and text difficulty was marginally significant, *F*(1, 58) = 3.81, *p* = 0.056, indicating that in general, insertions made by the dictation group were more sensitive to text difficulty than those made by the typing group.

#### 4.3.2. Deletions

**Figure 6:**
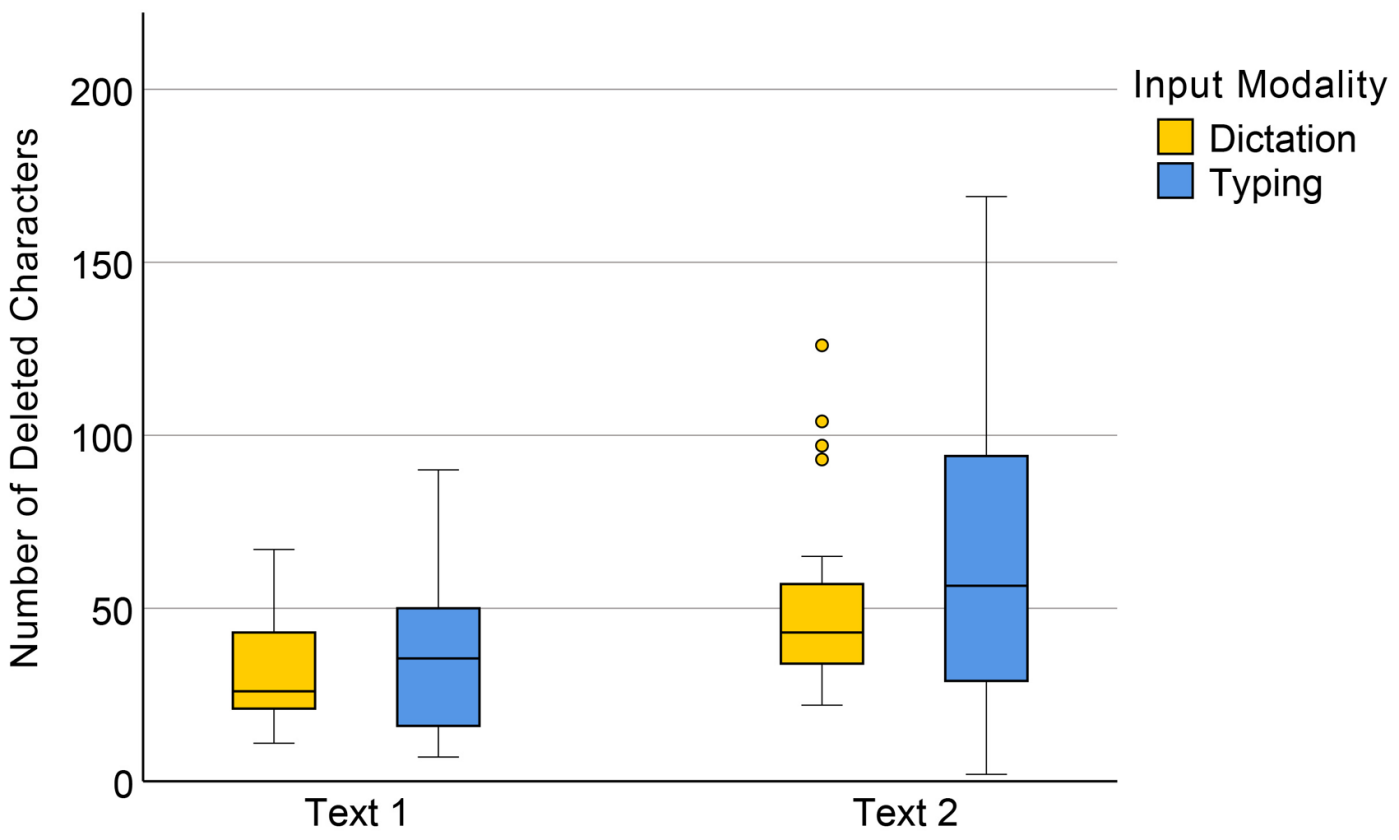
Numbers of deleted characters in dictation and typing conditions.

The data analysis showed no significant main effect of input mode on the number of deletions, *F*(1, 58) = 0.91, *p* = 0.345. However, there was a significant main effect of text difficulty, *F*(1, 58) = 42.29, *p* < 0.001, indicating that significantly more deletions were made during the translation of Text 2 (with a higher difficulty level) than Text 1 in both input conditions (see Figure 6). The interaction between input mode and text difficulty was non-significant, *F*(1, 58) = 0.68, *p* = 0.414.

### 4.4. Pause duration

**Figure 7:**
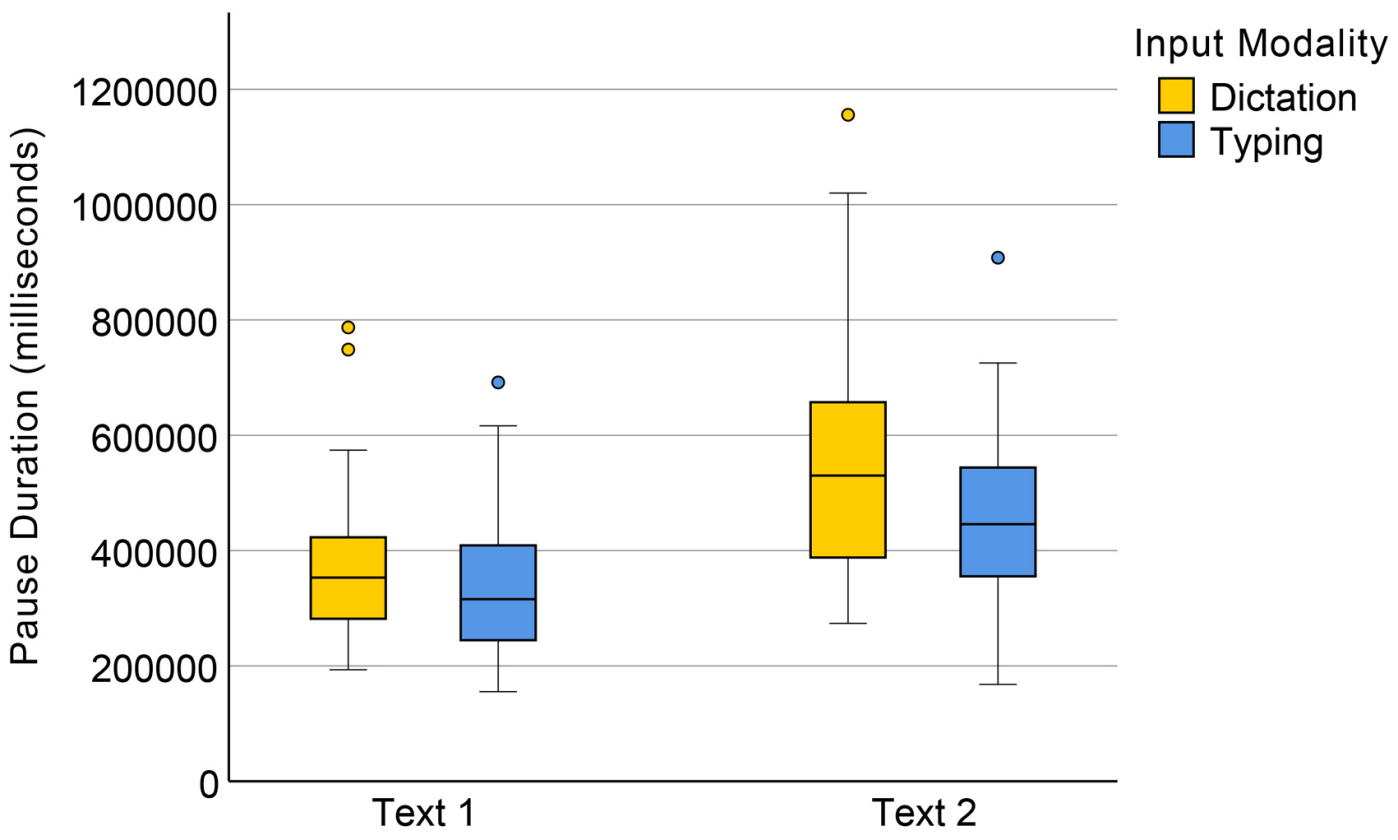
Duration of pauses in dictation and typing conditions.

The results of a Mann–Whitney U test showed that the difference in pause duration between the dictation group (Mdn = 416,999 ms) and the typing group (Mdn = 378116.5 ms) was marginally significant *U* = 1,440, *p* = 0.059 (see Figure 7). The results of a factorial ANOVA on the aligned and ranked data showed a significant main effect of text difficulty on pause duration *F*(1, 58) = 66.21, *p* < 0.001, but no significant interaction between input condition and text difficulty, *F*(1, 58) = 1.11, *p* = 0.296.

### 4.5. Ratings of text difficulty

A factorial ANOVA was conducted with input condition and text difficulty as the independent variables and difficulty rating as the dependent variable. The main effect of text difficulty yielded an *F* ratio of *F*(1, 58) = 76.83, *p* < 0.001, with the average difficulty ratings for Text 1 and Text 2 being 3.65 (SD = 1.02) and 4.84 (SD = 0.90) respectively. However, we failed to observe the main effect of input mode, *F*(1, 58) = 0.03, *p* = 0.867. The interaction effect was also non-significant, *F*(1, 58) = 1.13, *p* = 0.292.

### 4.6. Quality scores

A factorial ANOVA showed a significantly lower quality score for Text 2 than Text 1, *F*(1, 58) = 21.64, *p* < 0.001. The main effect of input condition was non-significant, *F*(1, 58) = 0.28, *p* = 0.599. We observed no significant interaction effect, *F*(1, 58) = 0.47, *p* = 0.494.

### 4.7. Results of the post-task questionnaire for the dictation group

In the dictation group, 20 out of 30 participants reported having experience with ASR tools, but only a small number of them used the tools frequently. Only two participants had tried ASR-assisted translation. When asked about their willingness to use ASR for translation in the future, 13 participants answered “yes,” while 17 answered “no.” The participants were also asked to identify the defects of Sogou Pinyin, the ASR tool used in the experiment. Their responses were grouped into the following categories:

1. Incorrect insertion of punctuation marks (15 mentions).
2. Insufficient recognition accuracy (13 mentions).
3. Inability to filter out vocal fillers (10 mentions).
4. Inadequate speed of speech-to-text conversion (3 mentions).
5. Sensitivity to background noise (2 mentions).

For the first problem concerning punctuation, participants noted that the ASR system tended to enter punctuation marks whenever the speaker paused, including when they merely paused to take a breath or to think rather than to signal the end of a sentence. On other occasions where the ASR system did segment the dictation properly, the added punctuation mark was often inappropriate. For example, the system might insert a period where there should have been a comma or a semicolon. In their criticisms about recognition accuracy, three participants reported issues with the recognition of homophones such as “的” and “地,” and three were dissatisfied with the recognition of proper nouns, such as names of people and countries.

## 5. Discussion

The present study examined the impact of ASR input on the efficiency and quality of translation among trainee translators. Specifically, we sought to investigate the performance of individuals utilizing ASR-assisted translation and evaluate whether this input mode influenced the efficiency and quality of their translations. The findings are discussed in the following sections.

### 5.1. Translators’ productivity and time allocation when dictating

In this study, we found that translation efficiency was not higher when using ASR input compared to conventional keyboard-and-mouse input. This result contradicts previous research (Désilets et al., 2008; Dragsted et al., 2011; Mees et al., 2013; Carl et al., 2016) which has found that ASR input leads to increased efficiency.

This discrepancy may be due to differences in the set-ups of translation workflow between our study and previous research. While most previous studies instructed participants to perform simple revisions using oral commands in the ASR system, our study examined the entire dictation process, including thorough revision of drafts. As a result, participants in our study aimed for high-quality translations, leading to longer revision time and overall task duration.

The limited increase in productivity observed in the dictation group can directly be attributed to their orientation and revision phases. While the dictation group’s drafting time was significantly shorter than that of the typing group, their orientation and revision times were longer. The revision phase occupied 58.23% of the dictation group’s total task duration, almost as much as the drafting phase in the typing group. The revisions in the dictation group were classified into four categories: wording, punctuation, misrecognitions, and filled pauses. The majority of the revision time was spent on rewording, specifically the detection and correction of inappropriate expressions.

Therefore, the amount of time saved may largely depend on the translators’ sight translation skills and experience, and there is a need for further developments of ASR systems to enhance the productivity of translators. While individual differences in translators’ interaction with ASR have been noted (Ciobanu, 2014), improving sight translation skills may lead to a reduction in revision time and shorter orientation time, resulting in drafts of higher quality (Li, 2014; Krapivkina, 2018). Training in sight translation can also improve the speed, accuracy, fluency, and adequacy of the process, reducing cognitive effort for the translator (Ho et al., 2020). In addition to sight translation skills, the experience of the translator may also impact their interaction with ASR (Ciobanu, 2014). Experienced translators tend to be more efficient, have shorter orientation and planning times, and allocate their time more strategically (Carl and Buch-Kromann, 2010; Lee, 2012).

### 5.2. Translation quality in the dictation mode

The results of the blind quality assessment of the translation outputs produced by the dictation group and the typing group indicated that working in dictation mode did not negatively impact translation quality, even for the more challenging translation task. These findings are consistent with those of previous studies (Dragsted and Hansen, 2009; Dragsted et al., 2011; Mees et al., 2013). One difference between this study and previous ones is that a thorough self-revision was conducted after participants completed the draft using ASR input, while only basic revisions were made in previous studies through oral commands to the ASR system. It appears that the impact of the translators’ skills on translation quality may have outweighed the differences in text production modes.

Previous research has suggested that if misrecognitions or other minor issues are left unaddressed when dictating translations using an ASR system, the quality of the final output will be reduced (Ciobanu, 2014, 2016). Our discussions with the evaluators following the quality assessment revealed that the quality issues in the dictation group and the typing group might have different causes. For instance, texts from the dictation group had more typos, punctuation errors, and inconsistencies in the names of people within the same paragraph. These are negative effects on translation quality potentially linked to ASR input.

As this study used a holistic approach to quality assessment, it is uncertain to what extent, if any, translation quality was impacted by the use of ASR for translation dictation. Future research may conduct more detailed analyzes of translation quality in ASR-assisted translation to further explore this issue.

### 5.3. Editing effort and cognitive effort in the dictation mode

Overall, the dictation group made fewer insertions and deletions than the typing group. These results align with those of Carl et al. (2016), who found that using ASR can reduce translators’ physical effort by eliminating the need for keyboard editing operations, despite the thorough revision required for dictated translations. However, our results regarding cognitive effort in dictation mode differ from those of Carl et al. (2016). While they found that English-Japanese translators experienced lower cognitive effort when dictating translations compared to typing them based on gazing and pause data, we observed longer pause durations in the dictation group but no significant difference in subjective ratings of text difficulty between the two groups. We also tested the correlation between pause duration and subjective ratings of text difficulty using Kendall’s tau coefficient, and the result was positive (*τ_b_* = 0.28, *p* < 0.001), indicating that the two measures were reliable. In addition, while Carl et al. (2016) suggested that total task time can be used as an indicator of cognitive effort, we found no significant difference between the dictation group and typing group in this regard. These results may be influenced by the translators’ experience or skills, as well as language-pair specific factors, such as the marked differences in syntactical and grammatical structures between English and Chinese, which may pose additional challenges for translators’ short-term memory compared to European language pairs (Agrifoglio, 2004).

In the post-task questionnaire, a surprising number of participants in the dictation group (17 out of 30) indicated that they would not like to use ASR support in future translation tasks. Some respondents mentioned that working with ASR was stressful. This aligns with the findings of Baghi and Khoshsaligheh (2019), who observed higher levels of stress, as indicated by increased blood pressure and heart rate in participants performing sight translation compared to written translation. These results suggest that further research is needed to better understand the cognitive aspects of ASR-assisted translation and translators’ interaction with speech technologies, particularly within the field of human-computer interaction.

### 5.4. Suggestions for customizing ASR tools for translation purposes

Based on the feedback from participants who expressed dissatisfaction with ASR support in the questionnaire and their revisions to the dictated drafts, we suggest the following improvements for ASR systems to be more effective in supporting translators.

First, ASR systems should offer users the option of dictating punctuation marks or utilizing automatic pause-based punctuation. This allows translators to avoid interrupting the flow of a sentence with an automatically generated punctuation mark when pausing during dictation. Alternatively, users can opt for the convenience of automatic pause-based punctuation if they prefer to save the effort of dictating punctuation marks.

Secondly, ASR systems should offer users the option to filter out filled pauses, such as “um” and “uh,” during transcription. These filler words can be a nuisance for translators and can potentially decrease the quality of translations. However, in certain translation tasks, such as character dialogs, translators may prefer to keep these fillers transcribed in order to maintain the authenticity of the original text. Therefore, providing users with the option to filter out or include filled pauses allows for greater flexibility and customization in the transcription process.

Thirdly, one way to improve the recognition accuracy of ASR systems is to utilize extensive terminology databases. In our experiment, a significant number of misrecognitions occurred with named entities, such as names of people and countries. By training the ASR tool to recognize these names, the recognition accuracy could be improved and the need for translators to make revisions could be reduced. Term bases can be especially helpful for experienced translators working on domain-specific documents that contain a high density of technical terms.

### 5.5. Limitations of the study and suggestions for future research

The present study has several limitations that should be acknowledged. Due to the COVID-19 pandemic and the related lockdowns, it was not possible to recruit professional translators for on-campus studies. As a result, this study only included translation trainees. Future research involving experienced professional translators, particularly those with experience using ASR tools, may yield different or more informative results. It would be valuable to explore the perspectives and experiences of professional translators in relation to ASR tools and their use in translation tasks.

When designing the study, internal validity was given priority over ecological validity. As a result, the experimental set-up differed from the normal working conditions of translators, which may have influenced the participants’ performance. To address this limitation, future research could consider allowing participants to use their own computers in their normal working environments. Additionally, it is worth noting that both between-subjects and within-subjects experimental designs have potential drawbacks. Between-subjects designs may be impacted by individual differences within and between groups, while within-subjects designs may be affected by learning effects or other changes that occur over the course of the study.

This study did not include eye-tracking data. Previous research has suggested that eye-tracking metrics, such as fixation duration, fixation counts, and pupil sizes, can be effective and reliable measures of cognitive load (Jakobsen, 2011; Hvelplund, 2017). Therefore, it may be beneficial for future research to incorporate such data in order to gain a deeper understanding of the cognitive processes involved in using ASR systems for translation tasks.

## 6. Conclusion

This study aimed to investigate the impact of ASR on translators’ performance. The results showed that ASR did not significantly increase productivity. While ASR assistance resulted in a reduction in drafting time compared to conventional keyboarding, it also required longer orientation and revision time. The revision phase took up the largest proportion of total task time in the dictation mode.

However, using ASR for drafting could potentially save translators typing time without compromising the quality of the translation output or increasing cognitive effort. The revisions of the dictation group suggest that sight translation skills may play a key role in increasing productivity and reducing cognitive effort. Translation trainees reported a mixed attitude toward the ASR-assisted mode. It is suggested that ASR tools could be customized for translators in terms of punctuation mark insertion, filled pause filtering, and expansion of terminology databases.

## Data availability statement

The raw data supporting the conclusions of this article will be made available by the authors, without undue reservation.

## Ethics statement

The studies involving human participants were reviewed and approved by Beijing Foreign Studies University. The patients/participants provided their written informed consent to participate in this study.

## Author contributions

LW and SS conceived the main idea of the study, discussed the project design and execution, and performed the statistical analyses. LW conducted the experiment and collected the data with the help of SS. LW wrote the first draft of the article and SS revised it. SS supervised the entire work. Both authors contributed to the article and approved the submitted version.

## Funding

This work was supported by the National Social Science Fund of China (grant number 19BYY115).

## Conflict of interest

The authors declare that the research was conducted in the absence of any commercial or financial relationships that could be construed as a potential conflict of interest.

## Publisher’s note

All claims expressed in this article are solely those of the authors and do not necessarily represent those of their affiliated organizations, or those of the publisher, the editors and the reviewers. Any product that may be evaluated in this article, or claim that may be made by its manufacturer, is not guaranteed or endorsed by the publisher.

## Footnotes

^1^ https://shurufa.sogou.com/

^2^ https://www.ieway.cn/evcapture.html

^3^ https://sites.google.com/site/centretranslationinnovation/tpr-db

^4^https://depts.washington.edu/acelab/proj/art/
